# An atypical orthologue of 6-pyruvoyltetrahydropterin synthase can provide the missing link in the folate biosynthesis pathway of malaria parasites

**DOI:** 10.1111/j.1365-2958.2007.06073.x

**Published:** 2007-12-18

**Authors:** Sabine Dittrich, Sarah L Mitchell, Andrew M Blagborough, Qi Wang, Ping Wang, Paul F G Sims, John E Hyde

**Affiliations:** Manchester Interdisciplinary Biocentre, Faculty of Life Sciences, University of Manchester 131 Princess Street, Manchester M1 7DN, UK

## Abstract

Folate metabolism in malaria parasites is a long-standing, clinical target for chemotherapy and prophylaxis. However, despite determination of the complete genome sequence of the lethal species *Plasmodium falciparum*, the pathway of *de novo* folate biosynthesis remains incomplete, as no candidate gene for dihydroneopterin aldolase (DHNA) could be identified. This enzyme catalyses the third step in the well-characterized pathway of plants, bacteria, and those eukaryotic microorganisms capable of synthesizing their own folate. Utilizing bioinformatics searches based on both primary and higher protein structures, together with biochemical assays, we demonstrate that *P. falciparum* cell extracts lack detectable DHNA activity, but that the parasite possesses an unusual orthologue of 6-pyruvoyltetrahydropterin synthase (PTPS), which simultaneously gives rise to two products in comparable amounts, the predominant of which is 6-hydroxymethyl-7,8-dihydropterin, the substrate for the fourth step in folate biosynthesis (catalysed by 6-hydroxymethyl-7,8-dihydropterin pyrophosphokinase; PPPK). This can provide a bypass for the missing DHNA activity and thus a means of completing the biosynthetic pathway from GTP to dihydrofolate. Supported by site-directed mutagenesis experiments, we ascribe the novel catalytic activity of the malarial PTPS to a Cys to Glu change at its active site relative to all previously characterized PTPS molecules, including that of the human host.

## Introduction

**Fig. 1:**
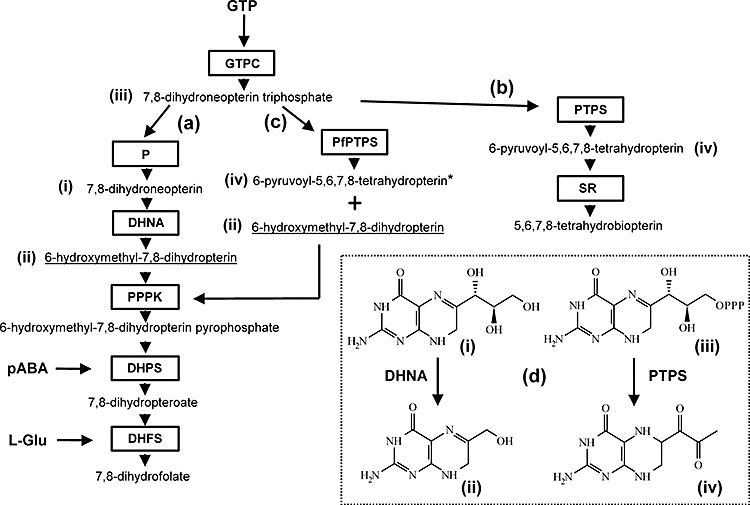
The conventional folate (a) and biopterin (b) biosynthetic pathways as found in (a) plants, bacteria and lower eukaryotes that are capable of *de novo* folate synthesis, and (b) in mammals and other organisms that utilize 5,6,7,8-tetrahydrobiopterin (BH_4_) as a cofactor. Certain organisms, such as some fungi, cyanobacteria and pseudomonads, possess both pathways. Pathway (c) involving the *P. falciparum* PTPS orthologue is demonstrated in this work and (d) shows the substrates (i), (iii) and products (ii), (iv) of conventional DHNA and PTPS enzymes respectively. Underlined product (ii) in pathways (a) and (c) is 6-hydroxymethyl-7,8-dihydropterin (6HMDP), the required substrate for PPPK. Asterisked product (iv) in pathway (c) was identified from its oxidation product (see text). Abbreviations: GTPC, GTP cyclohydrolase I; P, poorly defined phosphatase activity (thought in some systems to first involve loss of pyrophosphate then subsequent removal of the final phosphate); DHNA, dihydroneopterin aldolase; PPPK, hydroxymethyldihydropterin pyrophosphokinase; DHPS, dihydropteroate synthase; DHFS, dihydrofolate synthase; PTPS, pyruvoyltetrahydropterin synthase; SR, sepiapterin reductase.

Folate cofactors are essential molecules for all living organisms, required for the transfer of one-carbon units in a number of metabolic steps, including the key methylation of dUMP to give dTMP, an essential nucleotide for DNA synthesis. Most microorganisms can synthesize the required folates from the simple precursors GTP, *p-*aminobenzoate (pABA) and glutamate. Higher eukaryotes, with the exception of plants, are incapable of this and depend on a dietary intake of folate. Drugs targeting folate metabolism have long been used as highly successful antimicrobial and anticancer agents (Nzila *et al.*, 2005). The lethal human malaria parasite, *Plasmodium falciparum*, is capable of both *de novo* folate biosynthesis and salvage of pre-formed folate from the plasma of its human host, as demonstrated by radiolabelling studies with folate precursors and intact folates (Krungkrai *et al.*, 1989; Wang *et al.*, 2004a, b). Current evidence suggests that both routes are essential for sustained healthy parasite growth, although the reasons for this are unclear (Hyde, 2005). In other microorganisms (Bermingham and Derrick, 2002) and in plants (Cossins and Chen, 1997), the enzymic steps in the folate biosynthetic pathway are well established from decades of biochemical and genetic analysis. The purine ring system of GTP is rearranged by GTP cyclohydrolase I (GTPCH-I; EC 3.5.4.16) to that of a pterin (7,8-dihydroneopterin triphosphate; DHNTP), the three-carbon side-chain of which is subsequently cleaved to leave one carbon atom, after which pABA and glutamate are linked to the resulting pterin (6-hydroxymethyl-7,8 dihydropterin; 6HMDP) in successive steps involving 6-hydroxymethyl-7,8-dihydropterin pyrophosphokinase (PPPK or HPPK; EC 2.7.6.3), dihydropteroate synthase (DHPS; EC 2.5.1.15) and dihydrofolate synthase (DHFS; EC 6.3.2.12) [ Fig. 1, scheme (a)]. However, a long-standing mystery concerning this pathway in *P. falciparum* is the apparent lack of a gene encoding the enzyme required for the third step in this pathway, dihydroneopterin aldolase (DHNA; EC 4.1.2.25), the protein that removes two carbon atoms from the pterin side-chain as described above [Fig. 1, reaction (d) (i)–(ii)]. In contrast to the situation with all other folate biosynthetic pathway genes in *P. falciparum* (Brooks *et al.*, 1994; Triglia and Cowman, 1994; Lee *et al.*, 2001; Salcedo *et al.*, 2001), our attempts to clone a malarial *dhna* gene by degenerate oligonucleotide PCR based on conserved amino acid motifs in orthologues from other species were unsuccessful, and originally ascribed to low levels of conservation among such orthologues (e.g. *Escherichia coli*, *Bacillus subtilis*, *Pneumocystis carinii* and *Arabidopsis thaliana* DHNAs share identities of only *c.* 20–30%, with no contiguous sequences of conserved residues). However, upon subsequent completion of the genome sequence of *P. falciparum*, no *dhna* gene was apparent by blast searches (Gardner *et al.*, 2002a), nor has one been identified by similar analyses in other species of *Plasmodium* or in the related apicomplexan parasite *Toxoplasma gondii* that have been sequenced more recently. We therefore adopted several strategies, using both bioinformatics and biochemical assays, to further explore this observation and to identify how malaria parasites might cope with this apparent gap in their folate biosynthetic machinery. The results from such experiments demonstrate that *P. falciparum* possesses a thus-far unique variant of an enzyme associated in other organisms with the biosynthesis of tetrahydrobiopterin, which, via a novel mechanism involving a key amino acid alteration in the active site, can provide a bypass route to the substrate of the subsequent enzyme (PPPK) in the folate pathway.

## Results

### Bioinformatic searches for DHNA

Consistent with the initial analysis of the complete genome sequence of *P. falciparum* (Gardner *et al.*, 2002a), we found that psi-blast (Altschul *et al.*, 1997), pratt (Jonassen *et al.*, 1995) and similar search algorithms applied to the predicted complete proteome of *P. falciparum* failed to identify any statistically significant hits for a candidate DHNA enzyme. We therefore employed more powerful bioinformatics approaches based on secondary and tertiary structural queries using the programs 3d-pssm (Kelley *et al.*, 2000) and genthreader (GT) (McGuffin and Jones, 2003), which utilize different algorithms to predict structural analogues of a query sequence. This strategy exploited the fact that all DHNAs with determined three-dimensional crystal structures are members of a small family known as ‘tunnelling-fold’ (T-fold) proteins (Colloc'h *et al.*, 2000), established members of which are DHNA, GTPCH-I, urate oxidase (UO; EC 1.7.3.3), 6-pyruvoyltetrahydropterin synthase (PTPS; EC 4.2.3.12) and dihydroneopterin triphosphate epimerase (no EC number assigned) (Colloc'h *et al.*, 2002). All protein sequences longer than 50 amino acids (5893 sequences) predicted from the whole genome sequence by GlimmerM (Salzberg *et al.*, 1999; Gardner *et al.*, 2002b), developed for identifying coding sequences in *P. falciparum*, were processed through 3d-pssm and GT. Both identified unambiguously only two T-fold proteins in the parasite, GTPCH-I (3d-pssm‘expect’ value 1.33 × 10^−4^; GT ‘probability’ value 4 × 10^−8^) and a putative PTPS (values of 2.19 × 10^−12^ and 4 × 10^−5^ respectively) (Table 1). All other predicted proteins scored ≥ 1.18 in 3d-pssm and > 0.01 in GT. The 35 proteins falling between 1.18 and 20 in 3d-pssm and the 27 between 0.01 and 0.1 in GT (classed by the latter as ‘low confidence’) were examined individually, but none was a credible candidate for a DHNA orthologue. In addition to the statistical scores returned by the two programs, this conclusion was based on the absence in any candidate sequence of a GxxxxExxxxQ or closely related motif, found near the N-terminus in all known DHNAs (Fig. S1).

**Table 1:** Top six hits using the 3d-pssm program (Kelley *et al*., 2000) and searching the entire predicted *P. falciparum* proteome for putative T-fold protein types.

Gene locus in PlasmoDB^a^	T-fold protein type with highest match	3d-pssm *e*-value^b^	3d-pssm rank^c^	Annotation in PlasmoDB^a^
PFF1360w	PTPS	2.19 × 10^−12^	1	6-Pyruvoyl tetrahydropterin synthase, putative (PTPS)
PFL1155w	GTPCH-I	1.33 × 10^−4^	1	GTP cyclohydrolase I (GTPCH-I)
PFF0955c	DHNA	1.18	3	Hypothetical protein (with ribonuclease Rh-like InterPro domain)
PF14	DHNA	1.78	6	Ribosomal protein S3, putative
PFB0420w	UO	2.32	4	2C-methyl-d-erythritol 2,4-cyclodiphosphate synthase
PF10	DHNA	2.50	7	Hypothetical protein (with ribosomal L22 protein InterPro domain)

a From the *Plasmodium* sequence database at http://www.plasmodb.org/

b Matches with *e*-values greater than 1 are considered not to be statistically significant.

c Position in structural match table; this indicates whether the *P. falciparum* protein in question shows better matches to other structural families, as is the case for all but the top two entries above.

### Assay for DHNA activity in *P. falciparum* cell extracts

While strongly indicative of the absence of a DHNA-encoding gene in *P. falciparum*, the above bioinformatic methods all depend on a degree of similarity at the primary or secondary structural level. We therefore assayed for DHNA activity biochemically in *P. falciparum* cell extracts without any assumptions as to the possible nature of the protein. We initially coupled this reaction, using 7,8-dihydroneopterin (DHN) as the (normal) substrate, to the subsequent two enzymes (i.e. catalysing the fourth and fifth steps) in the folate biosynthetic pathway, PPPK and DHPS, in the form of the bifunctional PPPK-DHPS molecule cloned from *T. gondii* (Pashley *et al.*, 1997), and monitored the production of radiolabelled 7,8-dihydropteroate from [^14^C] *p*ABA [see Fig. S2, reaction scheme (a)]. This coupling strategy avoided the need for custom synthesis of labelled DHN for a direct assay. The protocol was verified using endogenous DHNA in extracts of a standard *E. coli* strain [BL21(DE3)] as a positive control. To establish that extracts from *P. falciparum* cultures were generally active for known enzymes, they were assayed in parallel for both endogenous PPPK-DHPS and serine hydroxymethyltransferase (SHMT; EC 2.1.2.1) activities, the latter by monitoring incorporation of ^14^C from labelled serine into 5,10-methylenetetrahydrofolate. As these activities are all involved in folate metabolism, we reasoned that if a DHNA were also present, it would be likely to exhibit a comparable level of activity. However, despite the expected behaviour of all positive controls, where we could readily measure the reaction products in both *E. coli* and malarial extracts from similar cell numbers (∼2.0 × 10^9^ to 1.0 × 10^10^), no DHNA activity could be detected in the latter preparations under the standard assay conditions used (Table 2).

**Table 2:** *Plasmodium falciparum* cell extracts have SHMT and PPPK-DHPS but not DHNA activities.

	SHMT^a^	PPPK-DHPS^b^	DHNA^b^
*P. falciparum*	4110 ± 100	930 ± 60	nd^c^
	(2.97 ± 0.07)	(0.66 ± 0.04)	(0)
*E. coli*	5110 ± 80	3800 ± 60	9830 ± 140
	(3.69 ± 0.06)	(2.70 ± 0.04)	(6.97 ± 0.10)

a Counts accumulated per 10 μl reaction aliquot after 1 h incubation and 1 h screen exposure.

b Counts accumulated per 10 μl reaction aliquot after 10 min incubation and 1 h screen exposure.

c No detectable counts above background.

All values corrected for counts recorded at time zero, normalized to equal numbers of cells. Means of three determinations ± SD; pmol equivalents in parentheses.

### Investigation of a putative malarial PTPS orthologue

**Fig. 2:**
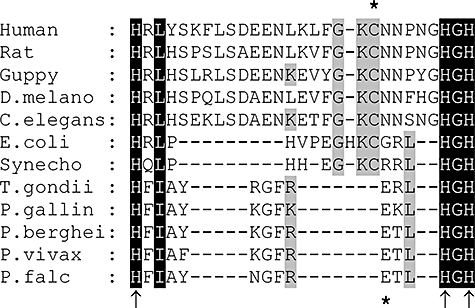
Aligned sequences of PTPS orthologues between the conserved His residues that co-ordinate the active site Zn^2+^ ion (arrows). The active site Cys residue conserved in all non-apicomplexan enzymes to date is marked by an asterisk above the sequences; the Glu residue proposed to act as nucleophile instead of Cys in *Plasmodium* is marked by an asterisk below (see also Fig. S3). Accession numbers for non-apicomplexan enzymes: human, Q03393; rat, P27213; guppy, Q90W95; *Drosophila melanogaster*, P48611; *Caenorhabditis elegans*, O02058; *Escherichia coli*, P65870; *Synechocystis*, Q55798. Gene loci for malarial sequences (http://www.plasmodb.org): *Plasmodium gallinaceum* (blast search); *Plasmodium berghei*, PB000950.03.0; *Plasmodium vivax*, Pv114505; *Plasmodium falciparum*, PFF1360w. The *Toxoplasma gondii* sequence was also determined from blast searching (http://www.toxodb.org).

With both the bioinformatics and biochemical data providing strong and complementary evidence that a conventional DHNA may be lacking in *P. falciparum*, we next explored the possibility that the DHNA step could be bypassed in malaria parasites, focusing on a protein putatively identified as a PTPS orthologue, which is encoded at the PFF1360w locus of the genome (http://www.plasmodb.org). The logical basis for this was twofold. First, this PTPS orthologue had been identified in the bioinformatics searches above as the only T-fold family protein in *P. falciparum* other than GTPCH-I (Table 1). Second, PTPS enzymes in other organisms use DHNTP as substrate, which is closely related to DHN, the substrate of DHNA [Fig. 1, reactions (d) (i)–(ii) and (iii)–(iv)], although at the primary sequence level, the DHNA and PTPS families are completely different, with no common motifs (Figs S1 and 3). In other organisms, including animals, fungi and certain bacteria, PTPS is the second enzyme in the pathway of tetrahydrobiopterin (BH_4_) synthesis, an essential cofactor for aromatic amino acid hydroxylases, glyceryl-ether monooxygenases and nitric oxide synthases (Thony *et al.*, 2000). Conventionally, PTPS converts DHNTP to 6-pyruvoyltetrahydropterin (PTP), which is then reduced by sepiapterin reductase (SR; EC 1.1.1.153) to BH_4_[ Fig. 1, scheme (b)]. Upon alignment of the putative *P. falciparum* PTPS with all other known PTPSs, a high level of conservation was apparent, including residues known from crystallographic studies of rat PTPS (Burgisser *et al.*, 1995; Ploom *et al.*, 1999) and *Caenorhabditis elegans* PTPS [Protein Data Bank (PDB) structures 1B66 and 2G64 respectively; http://www.rcsb.org/pdb/home/home.do] to be determinants for DHNTP binding (Fig. S3). However, a striking divergence in residues at the active site was evident (Fig. 2). In particular, the Cys residue (Cys-38/42/43 in *C. elegans*/rat/human PTPS; Fig. S3), which is completely conserved in all other known PTPSs, is absent from that of *P. falciparum*, of other *Plasmodium* species and of the related apicomplexan parasite *T. gondii*. The side-chain of this key residue ionizes to form the active nucleophile required for base-catalysed proton abstraction from the substrate (Burgisser *et al.*, 1995; Ploom *et al.*, 1999). Structural modelling using crystallographic data for the *P. falciparum* enzyme (PDB structure 1Y13) confirmed spatial differences between the malarial enzyme and those of other eukaryotes, and in particular, the presence of a Glu residue (Glu-38) in the former, closely similar in position and orientation to the active site Cys in the latter (Fig. 2 and Fig. S4). We thus hypothesized that, because of this change, the malarial PTPS enzyme may have different catalytic properties compared with conventional orthologues in other organisms.

### Activity of the *P. falciparum* PTPS orthologue

**Fig. 3:**
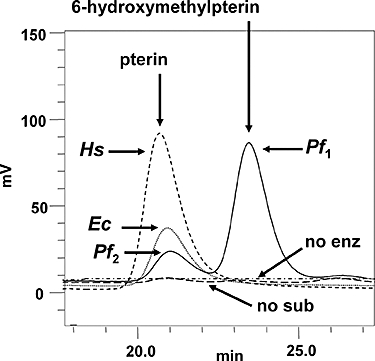
HPLC separation of pterin products arising from the reactions of the PTPS orthologues from *P. falciparum* (*Pf*_*1*_ and *Pf*_*2*_; solid line), *E. coli* (*Ec*; dotted line) and human (*Hs*; short-dashed line) using the normal substrate dihydroneopterin triphosphate (DHNTP), followed by oxidation to maximize fluorescence from the pterin ring system (Nichol *et al*., 1985). This process converts the products of the reaction 6-pyruvoyl-5,6,7,8-tetrahydropterin to pterin, and 6HMDP to 6-hydroxymethylpterin. No substrate (long-dashed line) and no enzyme (dot-dashed line) controls showed no fluorescence in these positions. Vertical arrows indicate the retention times of the pterin and 6-hydroxymethylpterin standards.

To test this hypothesis, we cloned the *P. falciparum ptps* gene into an *E. coli* expression system and purified the His-tagged product to near homogeneity. We then analysed this enzyme, together with His-tagged PTPS enzymes from *E. coli* and human as controls, for its ability to act on the natural substrate DHNTP. Pterin products were separated on high-performance liquid chromatography (HPLC) and monitored by the fluorescence of the pterin ring, which was oxidized prior to chromatography by acidic iodine to the fully conjugated form, to enhance fluorescence (Nichol *et al.*, 1985). Both the bacterial and human enzymes converted DHNTP to PTP as expected [an unstable compound that is detected after oxidation and consequent loss of the side-chain at position 6 as unsubstituted pterin (Woo *et al.*, 2002)]. However, the predominant peak of fluorescence (*Pf*_*1*_) arising from the malarial enzyme eluted at the same position as authentic 6-hydroxymethylpterin (the oxidized product of 6HMDP), together with a less intense, more rapidly eluted peak (*Pf*_*2*_) that coincided with the pterin derived from the bacterial and human enzyme reactions (Fig. 3). Control experiments using commercial 6HMDP confirmed that the hydroxymethyl side-chain, unlike the 1′, 2′-dioxopropyl side-chain of PTP, is stable to the ring oxidation conditions. Thus, in contrast to human and *E. coli* PTPS, which yield only the single expected product, the malarial enzyme gives rise to two different products. Although the ratios of these products were quite variable from run to run, by measuring the relative fluorescence intensities of equal concentrations of the 6-hydroxymethylpterin and pterin standards (1.72:1), we calculated from a series of assays (*n* = 13) that the two products are formed in broadly similar molar quantities, with the former exhibiting a slight predominance (1.48 ± 0.68 SD mol of 6-hydroxymethylpterin to 1 mol of pterin).

### Verification of PfPTPS production of 6HMDP by linkage to PPPK-DHPS reactions

**Fig. 4:**
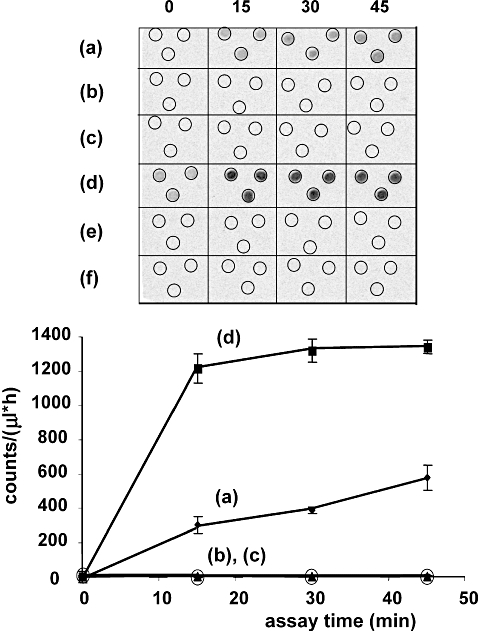
Confirmation of 6HMDP production by the *P. falciparum* PTPS (*Pf*) orthologue, but not by those from human (*Hs*) and *E. coli* (*Ec*). Products of the PTPS reaction were coupled to the next two enzymes in the pathway, PPPK and DHPS, in the form of recombinant bifunctional enzyme from *T. gondii*, and production of ^14^C-radiolabelled 7,8-dihydropteroate assayed in triplicate on a Typhoon imager [see Fig. S2, scheme (b), for the reaction set-up and more experimental detail]. Top, Typhoon image; bottom, plot of counts recorded after reaction times up to 45 min and expressed per 1 μl of reaction mix per hour of Typhoon screen exposure (mean ± SD, relative to zero time); (a) PfPTPS with DHNTP substrate, (b) HsPTPS with DHNTP substrate, (c) EcPTPS with DHNTP substrate, (d) 6HMDP, (e) no enzyme, but DHNTP substrate present, (f) PfPTPS, but no substrate present. In the bottom panel, negative controls (e) and (f) are omitted for clarity. In the positive control (d), commercial 6HMDP was provided as substrate for the PPPK step. One hundred counts on the ordinate are equivalent to 0.071 pmol product per μl of reaction mix. Qualitatively similar results were obtained when *P. falciparum* PPPK-DHPS was used in the coupled reaction instead of *T. gondii* PPPK-DHPS.

The retention time for 6-hydroxymethylpterin upon HPLC was well separated from those of other common pterins under our elution conditions and so was straightforward to identify. However, to confirm the assignment made for this slower eluting fluorescent product of the malarial enzyme from its retention time, we coupled the PTPS reaction to the PPPK and DHPS activities of both *T. gondii* and *P. falciparum*, as described above for DHNA [Fig. S2, scheme (b)]. The only known substrate for PPPK enzymes is 6HMDP (Brown, 1971), and both *P. falciparum* (Kasekarn *et al.*, 2004) and *T. gondii* (Smith, 2006) PPPK have been shown to process this compound in the normal way. Moreover, testing of a wide range of other pterins as potential substrates for malarial PPPK has yielded consistently negative results (S.L. Mitchell and J.E. Hyde, unpublished). In other organisms, 6HMDP is produced by DHNA in the third step of the conventional folate biosynthesis pathway [Fig. 1, scheme (a)]. If the PTPS orthologue from *P. falciparum* produces this compound, as the HPLC retention time indicated, then the product of the final enzyme in the coupled assay, radiolabelled dihydropteroate, should be detectable. A time-dependent accumulation of this product was indeed observed when the PfPTPS reaction was linked to either *T. gondii* PPPK-DHPS or the *P. falciparum* equivalent [ Fig. 4, sample (a)]. In contrast, when PTPS from either human or *E. coli* was linked to the PPPK-DHPS assay, no radioactive product was detected [Fig. 4, samples (b) and (c)], consistent with their producing only PTP in the conventional reaction, which is not a substrate for PPPK.

**Fig. 5:**
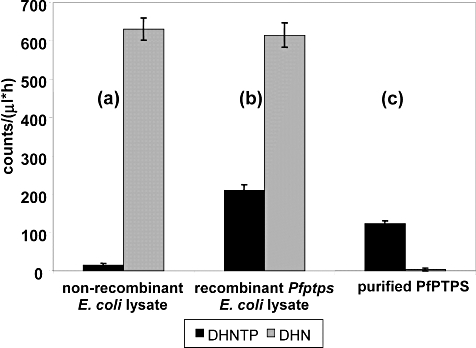
Formation of ^14^C-radiolabelled 7,8-dihydropteroate starting from either DHNTP (black) or DHN (grey) substrate (20 μM, 1 h incubation, 37°C) as recorded on the Typhoon imager (per 1 h screen exposure) from comparable amounts of (a) lysate from untransformed *E. coli* cells (and thus containing only EcDHNA) and (b) lysate from transformed *E. coli* cells overexpressing PfPTPS. The same experiment is repeated in (c) using affinity chromatography/ion-exchange-purified PfPTPS obtained from an equal volume of lysate as used in (b). One hundred counts on the ordinate are equivalent to 0.071 pmol product per μl of reaction mix.

To rule out the possibility of any contaminating *E. coli* DHNA activity producing 6HMDP from DHN that might be present as a minor component of our DHNTP preparations, further control experiments were necessary. First, non-recombinant *E. coli* lysate was incubated separately with equal concentrations of DHN and DHNTP for the standard period. A high level of DHNA activity was observed when DHN was provided as substrate, but the almost complete lack of reaction with DHNTP showed that < 2.5% of the latter had degraded by complete dephosphorylation to DHN [Fig. 5, sample (a)]. As expected, when recombinant *E. coli* lysate expressing PfPTPS was similarly tested, significant product could be made from both substrates [Fig. 5, sample (b)]. Critically, however, when affinity chromatography/ion-exchange-purified PfPTPS from the same lysate was assayed in this way, product was now only observed with DHNTP as the substrate [Fig. 5, sample (c)]. Together, these data (i) demonstrate that essentially all of the activity seen with DHNTP as substrate can be ascribed to the malarial gene product and (ii) exclude any contribution of *E. coli* DHNA carried over from the bacterial lysate acting on DHN derived from DHNTP breakdown to the production of 6HMDP in our assays. Thus, of the human, bacterial and parasite PTPS molecules tested, only the malarial orthologue displays the ability to produce 6HMDP, which it synthesizes specifically from DHNTP, and can thereby provide the bypass to PPPK necessitated by the apparent lack of a DHNA activity in this organism.

### Site-directed mutagenesis to explore the role of Glu-38

**Fig. 6:**
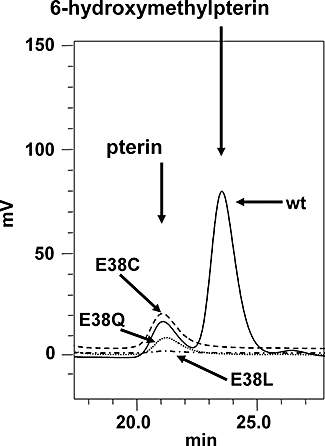
Effect of mutation of the Glu-38 residue in the *P. falciparum* PTPS orthologue on the reaction products. HPLC separated pterin products arising from the reactions of the wild type (solid line), E38C variant (dashed line), E38Q variant (dotted line) and E38L variant (dot-dashed line) using dihydroneopterin triphosphate (DHNTP) as substrate, followed by processing and calibration as for Fig. 3.

From the primary sequence (Fig. 2) and structural (Fig. S4) alignments of the *P. falciparum* PTPS orthologue with those of other organisms, we hypothesized that the substitution of a glutamate residue (Glu-38) in *P. falciparum* for the active-site cysteine residue of all other known PTPS molecules was likely to be a major factor in the novel ability of the malarial enzyme to produce 6HMDP from DHNTP, particularly as no other residue in this region has an ionizable side-chain capable of acting as a nucleophile for base catalysis. To investigate this experimentally, site-directed mutagenesis of the wild-type *Pfptps* clone was carried out to give a series of mutants, Glu38Cys, Glu38Gln and Glu38Leu. The rationale for these modifications was (i) substitution with the conventional Cys residue into the active site of the malarial molecule, (ii) substitution with the closely related residue (Gln) that retains the polar carbonyl group in the side-chain, but lacks the acidic proton whose dissociation renders the side-chain of Glu negatively charged at physiological pH, and (iii) substitution with a completely apolar side-chain (Leu) of comparable volume to that of Glu. The three mutant clones expressed soluble recombinant proteins at levels comparable to the wild type in *E. coli*, and these products all gave circular dichroic (CD) spectra closely similar to the wild type and characteristic of properly folded proteins (data not shown). Purified preparations of the four PTPS variants were assayed in parallel at equal concentrations after the standard incubation with DHNTP as substrate and the pterin products monitored by their fluorescence after HPLC separation (Fig. 6). As expected, the Glu38Leu mutant, lacking any nucleophilic centre in the side-chain, gave no detectable product, whereas the Glu38Cys mutant gave a clear peak of conventional pterin product, but importantly, no 6HMDP. This was also observed for the Glu38Gln mutant, albeit at a significantly reduced level. Only the wild-type enzyme yielded 6HMDP as well as the pterin peak. These data demonstrate the critical role of the Glu-38 side-chain in the production of the PPPK substrate.

## Discussion

Most microorganisms (Bermingham and Derrick, 2002) and plants (Cossins and Chen, 1997) synthesize folate *de novo* via a well-defined pathway involving GTPCH-I, DHNA, PPPK, DHPS and DHFS [Fig. 1, scheme (a)]. The predicted functions of all but one of these enzymes, DHNA, that are also expected to be present in *P. falciparum*, have been confirmed by biochemical assays on parasite-derived cloned gene products [GTPCH-I (P. Wang and J.E. Hyde, unpublished); PPPK (Kasekarn *et al.*, 2004); DHPS (Triglia *et al.*, 1997); DHFS (Salcedo *et al.*, 2001)]. Here, we were unable to detect DHNA activity in *P. falciparum* extracts that were demonstrably active in other enzymes of folate metabolism, although we cannot exclude the formal possibility of a parasite variant that is inactive under our standard assay conditions, in which DHNA is readily detected from *E. coli* lysates. However, these biochemical data are consistent with the uniformly negative bioinformatic searches for a DHNA-encoding gene using a range of algorithms that depend on similarities at either the primary sequence, or secondary/tertiary structural, levels. With respect to its component enzymes, *P. falciparum* is thus unusual in apparently lacking DHNA in an otherwise complete pathway that is essentially identical to that found in other folate-producing organisms. In addition, we demonstrate that, in the apparent absence of such a gene, the parasite encodes an unusual member of the PTPS family, which is a viable alternative to DHNA capable of synthesizing the 6HMDP required as substrate for PPPK from DHNTP. Moreover, the direct experimental coupling of this reaction to *P. falciparum* PPPK-DHPS to produce dihydropteroate now provides the basis for a complete definition of a pathway for folate biosynthesis in this organism [Fig. 1, scheme (c)]. We also note that deployment of the PfPTPS orthologue in the synthesis of 6HMDP would eliminate the need for a separate phosphatase activity to act on DHNTP, as depicted in the second step of the conventional pathway in Fig. 1, scheme (a). Microarray analyses (Llinas *et al.*, 2006) show that the gene encoding PfPTPS (PFF1360w) is expressed across the blood stages of all parasite lines investigated with a shallow peak at 18–22 h post invasion, corresponding to the early to mid-trophozoite stages. This resembles the patterns seen for the other folate biosynthesis genes, all of which peak during the 20–30 h phase of the 48 h asexual cycle (http://www.plasmodb.org and Nirmalan *et al.*, 2002; Llinas *et al.*, 2006). Interestingly, not only is PfPTPS capable of providing the PPPK substrate, it simultaneously generates a comparable amount of the normal PTP product of this class of enzyme, at least under our *in vitro* conditions. This suggests a possible dual role for PTPS in parasite pterin metabolism, and may relate to the discovery of a *P. falciparum* pterin 4a-carbinolamine dehydratase (PCD; EC 4.2.1.96) with conventional pterin recycling activity (Wang *et al.*, 2006). However, such a second role for PfPTPS may not lie in the standard BH_4_ synthesis pathway [Fig. 1, scheme (b)] because we detect little or no BH_4_ in parasite extracts, whereas 6HMDP is readily observed (P. Wang and J.E. Hyde, unpublished).

Despite significant primary sequence variation, all bacterial and eukaryotic PTPSs characterized to date preserve the key Cys residue in their active sites (Fig. 2), and we show here that, unlike the malarial enzyme, neither *E. coli* nor human PTPS is capable of producing 6HMDP from DHNTP (Figs 3 and 4). We ascribe this reaction to the replacement in *P. falciparum* of the Cys residue with Glu-38, whose side-chains in our models occupy a very similar spatial position and orientation relative to the active site (Fig. S4). That substitution of Glu-38 for Cys is the critical evolutionary modification of the malarial enzyme is strongly supported by our site-directed mutagenesis experiments, where reverse engineering of the wild-type PfPTPS to place Cys in the active site abolishes the production of 6HMDP and causes the enzyme to behave as a conventional PTPS molecule (Fig. 6). The shift to a Glu residue in the natural malarial enzyme replaces the single nucleophilic centre of Cys with negative charge distributed over the two side-chain oxygens of Glu, which could explain our observation of two products generated in comparable quantities by the parasite orthologue (see Fig. S5 and legend for a proposed mechanism). This hypothesis could be explored further by crystallography of the wild type and three mutant versions of PfPTPS with bound substrate/product or analogues, which would also extend the only structural data of PfPTPS currently available, where the wild-type enzyme is bound to biopterin in PDB structure 1Y13.

A previous study has indicated that disruption of the folate biosynthetic pathway by gene deletion in this haploid organism is apparently lethal, even in the presence of exogenous (salvageable) folate, and thus unlikely to be a feasible route to confirm the *in vivo* role of PfPTPS in this pathway (Wang *et al.*, 2004b). Moreover, no specific inhibitors of PTPS molecules have yet been reported that could be utilized to this end. However, the bioinformatic and biochemical evidence we report here is consistent with the absence of a conventional DHNA activity, while revealing the presence of an unusual apicomplexan PTPS orthologue whose product provides the necessary substrate for PPPK. This suggests that *P. falciparum* and related parasite species have evolved a novel and unexpected route of folate biosynthesis and that the structural and biochemical differences between the malarial and mammalian PTPS orthologues identified here might be exploitable in the search for new parasite targets within this critical pathway. However, given the major importance of the BH_4_ synthesis pathway in humans, any candidate inhibitors would need to show a marked discrimination between the host and parasite molecules.

## Experimental procedures

### DHNA, PPPK, DHPS and SHMT activities

DHPS activity was measured by the incorporation of [ring-^14^C] *p*ABA into DHP, as previously described (Aspinall *et al.*, 2002); DHNA and PPPK were monitored by coupling their activities to that of DHPS, using the appropriate substrates: 6HMDP for PPPK activity and 7,8-dihydroneopterin for DHNA activity (Schircks, Switzerland) [Fig. S2, scheme (a)]. Standard reactions (100 μl) contained 20 μM pterin substrate, 0.1 M Tris-HCl (pH 8.0), 5 mM DTT, 10 mM MgCl_2_, 20 μM ATP (for DHNA and PPPK activities), 20 μM [^14^C] *p*ABA (specific activity 58 mCi mmol^−1^; Moravek, California) and enzyme. To couple the DHNA reaction, mixes were supplemented with 2 μg of recombinant PPPK-DHPS from *T. gondii* (Aspinall *et al.*, 2002) or from *P. falciparum* (Triglia *et al.*, 1997), as appropriate. The reaction was incubated at 37°C for 1 h, together with negative controls lacking either enzyme or substrate. Aliquots (25 μl) were removed and reactions stopped by EDTA added to 2.5 mM. Aliquots were then spotted onto DE-81 paper (Whatman, UK) pre-soaked in 2.5 mM EDTA (pH 8.0) and air-dried. Unincorporated [^14^C] *p*ABA was removed by washing for 1 h in 80 mM NaCl, 0.1 M Tris-HCl (pH 7.9) at room temperature (3 × 500 ml). The radioactivity incorporated into [^14^C] DHP on the dried paper was detected on a Typhoon scanner (Amersham, UK) after ∼24 h exposure. Counts were quantified using the ImageQuant program and corrected for background counts seen in negative controls and thus are expressed as absolute product counts. SHMT activity was measured by incorporation of ^14^C from serine to 5,10-methylenetetrahydrofolate using a modified protocol (Smith, 2006) based on Geller and Kotb (1989). The assay mixture (100 μl) contained 50 mM Tris-HCl (pH 8.0), 3 mM DDT, 0.25 mM pyridoxal 5′-phosphate (Sigma, UK), 2.5 mM EDTA, 2 mM tetrahydrofolate (Schircks, Switzerland), 0.024 mM [3-^14^C] serine (57 mCi mmol^−1^, Moravek, CA), 0.2 mM unlabelled serine and enzyme, and radiolabelled product counts determined as above, except that the chromatography paper was washed in running dH_2_O for 2 h to remove unconverted ^14^C-serine. Counts detected in this way were converted to picomoles of product by comparison with standard curves generated by directly imaging known quantities of the radiolabelled substrates and assuming equimolar conversion to product during the enzyme reactions.

### Cell extracts

*Plasmodium falciparum*-infected erythrocytes were saponin-lysed and the harvested parasite pellets (∼1–5 × 10^10^ parasites) re-suspended in 500 μl of Reporter Gene Lysis Buffer in the presence of Complete C Protease Inhibitor Cocktail, 1 U DNase and 1 U RNase (all reagents from Roche, Switzerland) and incubated at room temperature with gentle agitation for 15 min. The resultant lysates were centrifuged (14 000 *g*, 30 min) at 4°C and the soluble fraction decanted and concentrated to 200 μl, from which 40 μl was used per assay. *E. coli* pellets from cultures of known density were lysed using BugBuster detergent (Novagen, UK) according to the manufacturer's guidelines in the presence of Complete C Protease Inhibitor Cocktail, 1 U DNase and 1 U RNase. The soluble fraction was decanted into a fresh tube after centrifugation and reduced to 1/50 of the volume of initial culture, from which 40 μl was used per assay.

### Cloning and expression of the *P. falciparum ptps gene*

The intronless *ptps* gene from *P. falciparum* genomic DNA (K1 strain) was PCR amplified using primers tailored to the Ek/LIC (Novagen) expression system: GACGACGACAAGATGATGAAAGAGGAAACCCTAAATTCAG (forward) and GAGGAGAAGCCCGGTTTATATATATTTGTGGACTATGGCCTTTTGTG (reverse). The PCR product was extracted after gel separation using Zymoclean (Zymo Research, USA) and cloned into the pET-46 Ek/LIC vector (Novagen, UK) to produce the plasmid Pfptps-pET46 by ligation-independent cloning using T4 polymerase.

### Purification of recombinant PTPS

*Escherichia coli* strain Rosetta2(DE3)pLysS (Novagen, UK) was transformed with Pfptps-pET46, induced by 1 mM IPTG at an OD_600_ of 0.6–1.0, and the cells were grown for a further 5 h at 30°C, harvested by centrifugation and lysed using BugBuster (Novagen, UK). The PfPTPS N-terminal His-tagged fusion protein in the soluble crude extract was purified by absorption onto a column of Ni-NTA resin (Qiagen, UK), washing with 20 mM imidazole in 300 mM NaCl, 50 mM NaH_2_PO_4_, pH 8.0, elution with 250 mM imidazole in the same buffer, dialysis into 100 mM Tris-HCl (pH 8.0), 2 mM DTT and storage in 10% glycerol at −80°C until further use. His-tagged PTPSs from *E. coli* and human were produced in the same way from pET-28a-based clones pET-ePTPS and pET-hPTPS respectively (Woo *et al.*, 2002). Further purification of the enzyme was carried out on a Resource Q ion exchange column (GE Healthcare, UK), binding in 50 mM Tris (pH 8.0), 5 mM DTT, 25 mM NaCl, and eluting in the same buffer except with 1 M NaCl.

### Assay of PTPS activity

PTPS activity was assayed according to Lee *et al.* (1999) with minor modifications. Assays were performed in 100 mM Tris-HCl (pH 8.0), 10 mM MgCl_2_, 9 μM DHNTP, 2 mM DTT with 1–2 mg ml^−1^ purified PTPS in a total volume of 50 μl for 1 h at 37°C. The reaction mixture was treated with 100 μl of acidic iodine solution (2% KI/1% I_2_ in 1 M HCl) for 1 h in the dark to oxidize the pterin ring and thereby maximize fluorescent intensity (Nichol *et al.*, 1985). After centrifugation, excess iodine in the supernatant was reduced to iodide by 50 μl of 5% ascorbic acid, the sample passed through a 0.2 μm Micro-Spin filter (Alltech Associates, UK) and 20 μl analysed for pterins by HPLC on a Dionex Summit system. A reverse-phase column, Adsorbosil C18, 10 μm, 250 × 4.6 mm internal diameter (Alltech Associates, UK), was used with a mobile phase of 10 mM sodium phosphate, pH 6.0, at a flow rate of 1 ml min^−1^ for 40 min. Fluorescence excitation and emission wavelengths were set to 350 nm and 450 nm, respectively, and areas under peaks used to calculate product ratios. Retention times were compared with pterin standards obtained from Schircks, Switzerland. Absolute retention times were critically dependent on the condition of the column from previous usage, so all samples and standards for a given experiment were run in immediate succession.

DHNTP substrate was produced from GTP in a separate reaction using *Synechocystis* GTPCH-I as described (Lee *et al.*, 1999). DHNTP concentration was estimated from its absorbance at 330 nm using an extinction coefficient of 6300 M^−1^ cm^−1^ (Bracher *et al.*, 1998). Aliquots of the DHNTP reaction mixture were frozen at −80°C and used once for PTPS enzyme assays. The coupled PTPS-PPPK/DHPS assay was performed as described above for DHNA assays with minor modifications [Fig. S2, scheme (b)]. The reaction (200 μl) was performed in 100 mM Tris-HCl (pH 8.0), 5 mM DTT, 20 μM DHNTP, 10 mM MgCl_2_, 20 μM ATP, 20 μM [^14^C] *p*ABA, 1 mg ml^−1^ purified PTPS and 7.5 μg ml^−1^ TgPPPK/DHPS or 3 mg ml^−1^ of concentrated lysate from cells expressing PfPPPK/DHPS. Reactions were incubated at 37°C for 1 h. The positive control contained 20 μM 6HMDP (Schircks, Switzerland) instead of PTPS enzyme and substrate.

### Site-directed mutagenesis of the *Pfptps* gene

Three oligonucleotides were designed to mutate the codon for Glu-38 in the ‘FRETLH’ motif of the putative active site of the *P. falciparum ptps* gene (Fig. 2). GAA (Glu) was mutated to CTT (Leu) with 5′-TACAATGGTTTTCGActtACCTTACATGGTCATA, to CAG (Gln) with 5′-TACAATGGTTTTCGAcagACCTTACATGGTCATA and to TGC (Cys) with 5′-TACAATGGTTTTCGAtgcACCTTACATGGTCATA. Mutagenesis was carried out with the GeneEditor *in vitro* site-directed mutagenesis system (Promega, UK) on the expression plasmid PfPTPS-pET46 described above. All inserts were completely sequenced to verify the desired alterations and ensure that no others had been introduced.

## Supplemental material

This material is available as part of the online article from:

http://www.blackwell-synergy.com/doi/abs/10.1111/j.1365-2958.2007.06073.x

(This link will take you to the article abstract).
