# Supplementary material for: An atypical orthologue of 6-pyruvoyltetrahydropterin synthase can provide the missing link in the folate biosynthesis pathway of malaria parasites
Source: Mol Microbiol. 2007 Dec 18;67(3):609–18. doi: 10.1111/j.1365-2958.2007.06073.x (PMC2229834; doi:10.1111/j.1365-2958.2007.06073.x)
Supplement: Supplementary file 1 [file mmi0067-0609-SD1.pdf]

Supplementary material for:

An atypical orthologue of 6-pyruvoyltetrahydropterin synthase can provide the missing link in the folate biosynthesis of malaria parasites

Sabine Dittrich, Sarah L. Mitchell, Andrew M. Blagborough, Qi Wang, Ping Wang, Paul F. G. Sims and John E. Hyde

DHNA sequences

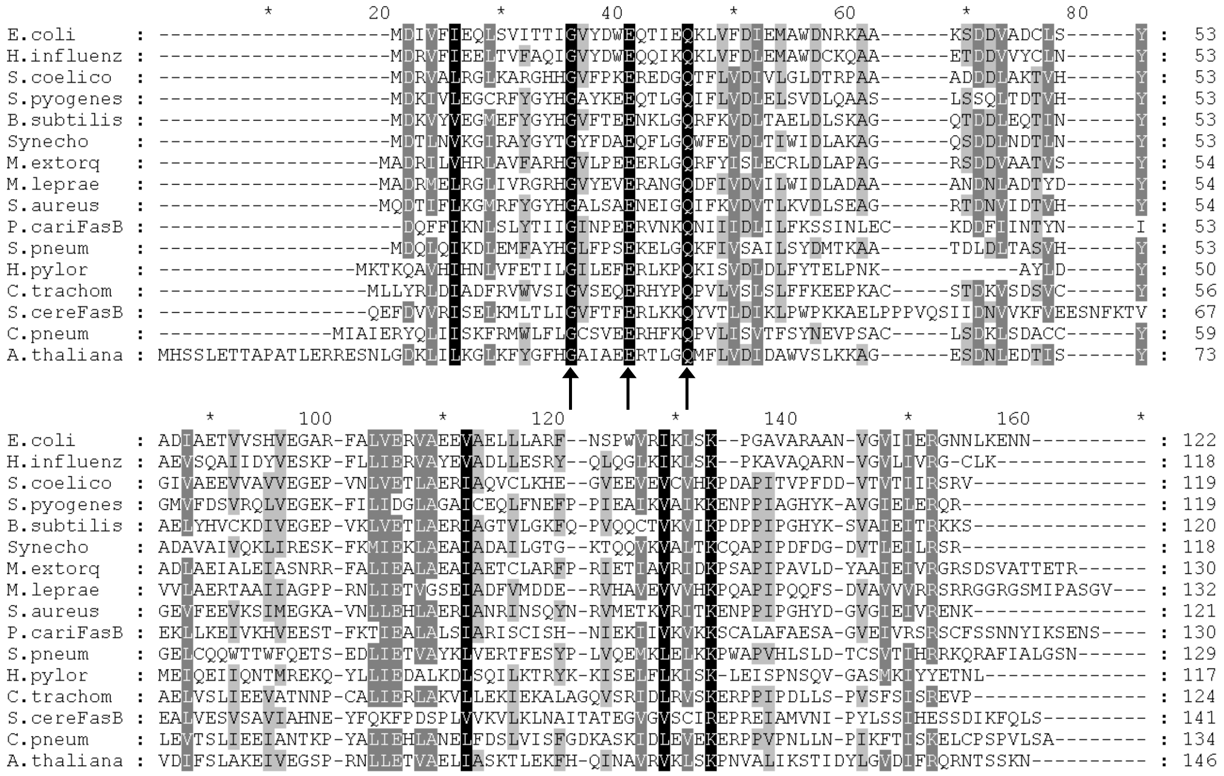

Fig. S1

Aligned sequences of 16 representative DHNA orthologs showing the GxxxxExxxxQ motif (arrows) conserved almost completely in all known members of this enzyme type. In descending order with accession numbers: *Escherichia coli*, P0AC16; *Haemophilus influenzae*, P46362; *Streptomyces coelicolor*, Q9X810; *Streptococcus pyogenes*, P0C0G5; *Bacillus subtilis*, P28823; *Synechocystis sp.*, P74342; *Methylobacterium extorquens*, P71513; *Mycobacterium leprae*, O69529; *Staphylococcus aureus*, Q6GJF6; *Pneumocystis carinii* FASB domain, P29251; *Streptococcus pneumoniae*,P22291; *Helicobacter pylori*, AAD06984; *Chlamydomonas trachomatis*, O84620; *Saccharomyces cerevisiae* FASB domain, P53848; *Chlamydia pneumoniae*, Q9Z7E9; *Arabidopsis thaliana*, Q9SF23.

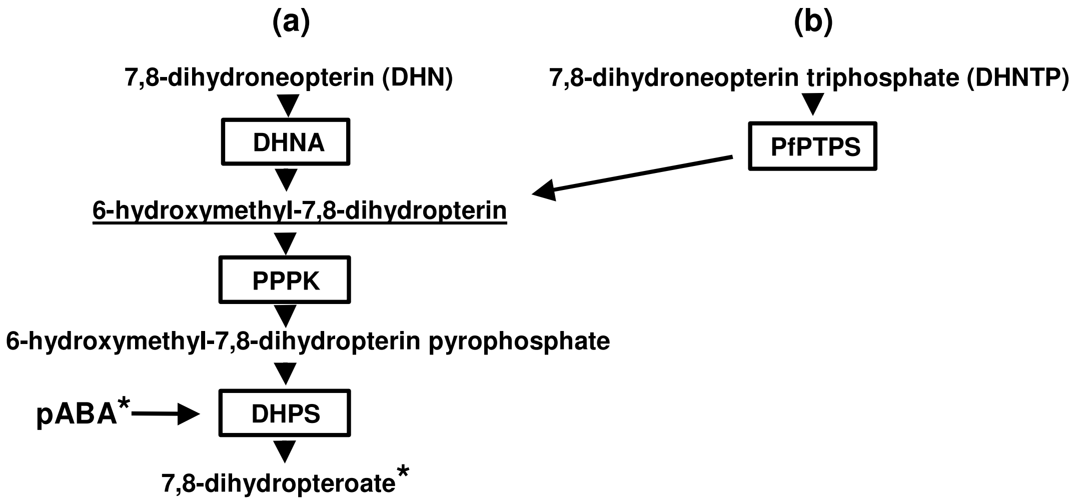

Fig. S2

Reaction schemes for assay of (a) DHNA and (b) PfPTPS activities. The appropriate substrate (DHN or DHNTP) is added to the enzyme sample and the production of the key intermediate 6-hydroxymethyl-7,8-dihydropterin (6HMDP; underlined) monitored by coupling to the PPPK and DHPS reactions, provided by addition of either bifunctional PPPK-DHPS from *Toxoplasma gondii* (Aspinall *et al.*, 2002) or *Plasmodium falciparum* (Triglia *et al.*, 1997). Positive reactions were detected by the incorporation of [^{14}C] *p*ABA into [^{14}C] 7,8-dihydropteroate (asterisks) and quantitation of the latter after ion-exchange paper chromatography on a Typhoon imager (Amersham, UK). For assay of PPPK-DHPS activity in parasite cell extracts, 6HMDP was added as substrate and supplementary PPPK-DHPS enzyme omitted.

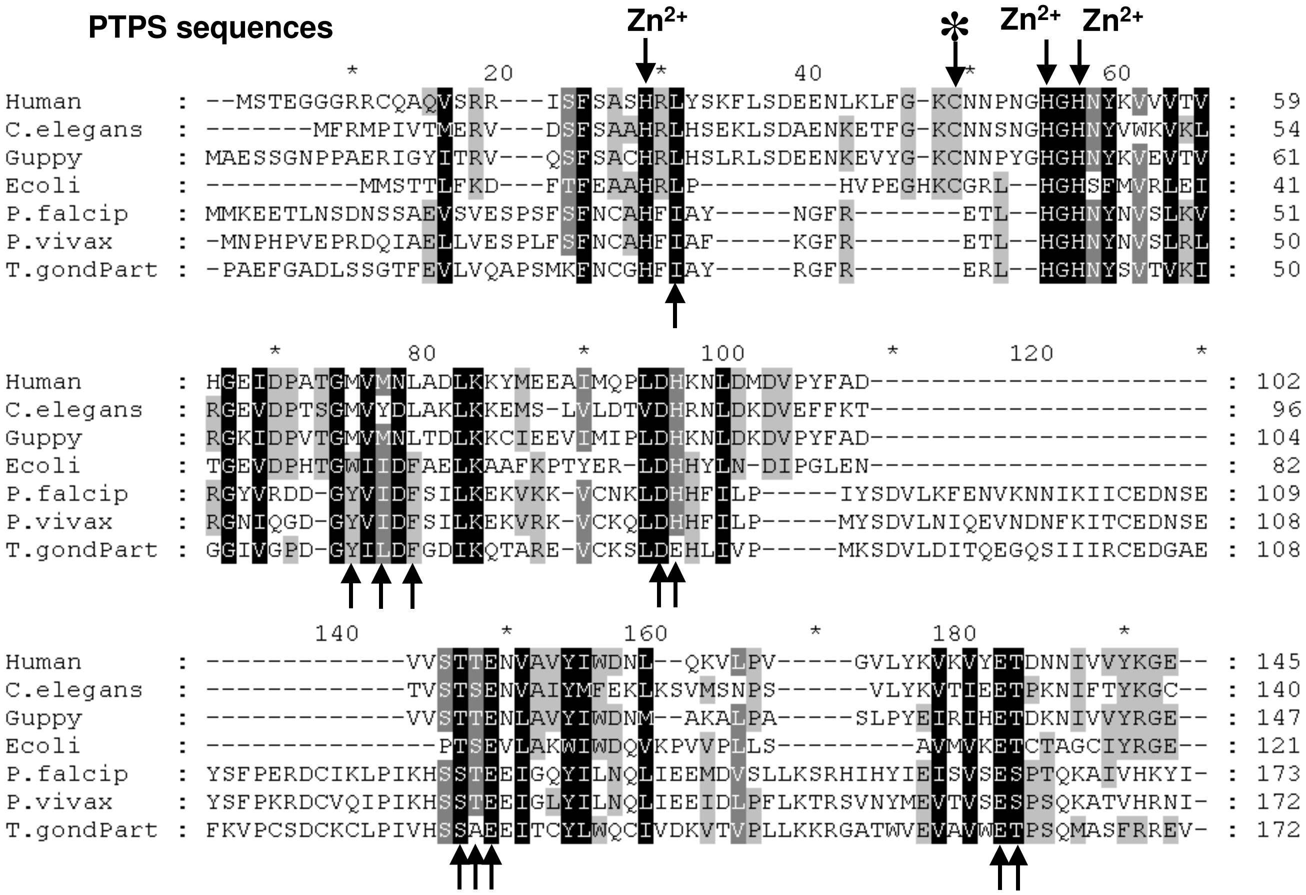

Fig. S3

Aligned sequences of selected PTPS orthologs. Downward arrows indicate the three completely conserved His residues that coordinate the active site Zn^{2+} ion and the active site Cys residue (large asterisk) conserved in all non-apicomplexan enzymes to date. Upward arrows indicate residues identified from crystallography (Burgisser *et al.*, 1995; Ploom *et al.*, 1999) to be important in substrate binding and catalysis. Accession numbers for non-apicomplexans: human, Q03393; *C. elegans*, O02058; guppy, Q90W95; *E. coli*, P65870. Gene loci for malarial sequences (www.plasmodb.org): *P. falciparum*, PFF1360w; *P. vivax*, Pv114505. The *T. gondii* sequence was determined from BLAST searching (www.toxodb.org) and is part of a much longer ORF extending towards the N-terminus; the section shown commences close to the start points of other PTPSs as aligned.

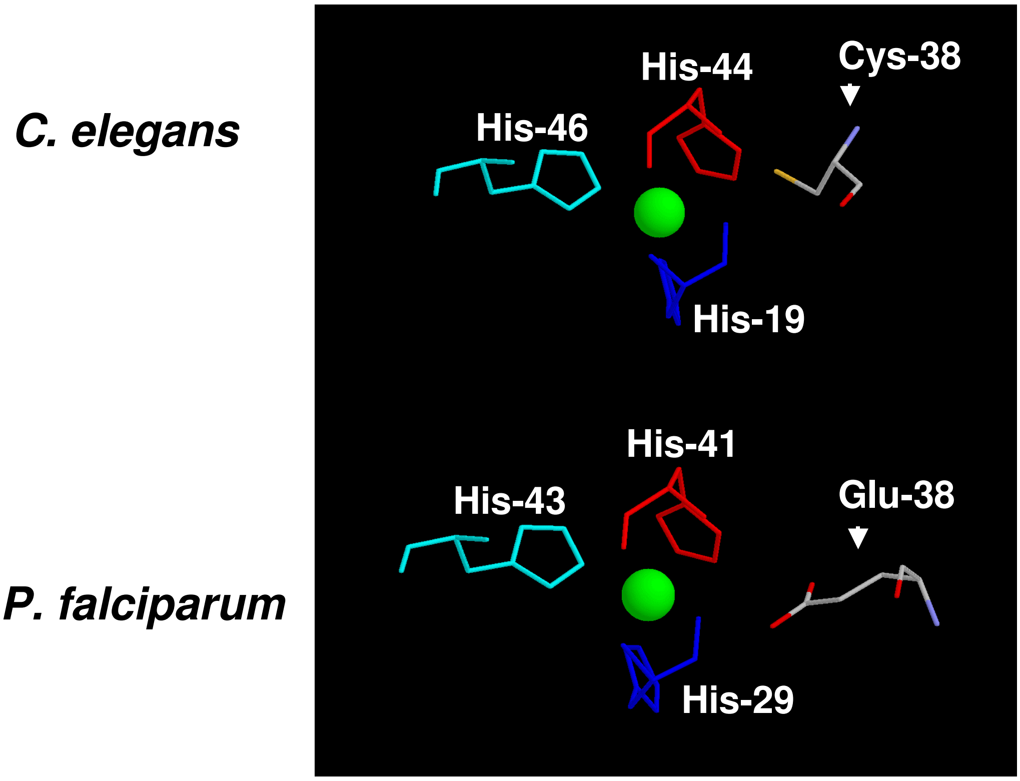

Fig. S4

Spatial comparison of the active site regions of *C. elegans* and *P. falciparum* PTPS molecules using the 3 x His-coordinated Zn^{2+} ion (green sphere) as reference point, showing the relative occupation in space of the active site nucleophile Cys-38 in *C. elegans* PTPS and the proposed nucleophile Glu-38 in PfPTPS. Distances of the nucleophilic centres from the Zn^{2+} ion are measured at 0.455 nm for the S atom (orange) of CePTPS Cys-38, and 0.413 and 0.518 nm (average 0.465 nm) for the two side chain O atoms (red) of PfPTPS Glu-38. Models are derived from the crystallographic coordinates of *C. elegans* PTPS and of the *P. falciparum* enzyme (PDB structures 2G64 and 1Y13, respectively; www.rcsb.org/pdb/home/home.do).

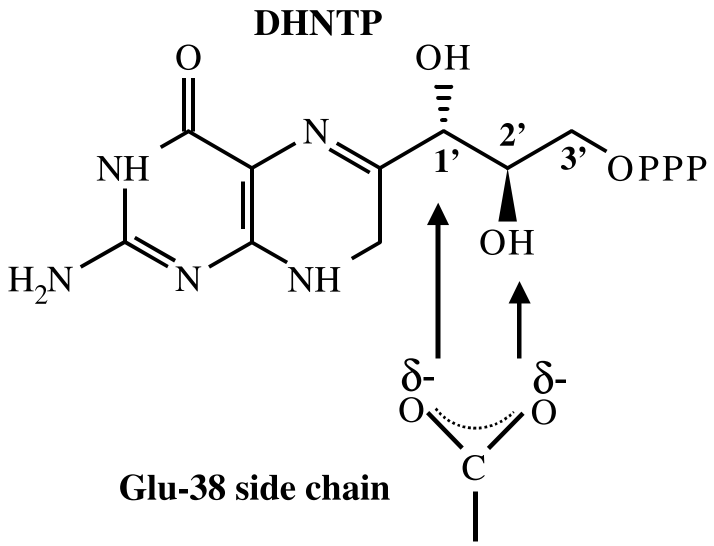

Fig. S5

Proposed nucleophilic attack of the Glu-38 residue of *P. falciparum* PTPS on the side chain of the DHNTP substrate leading to two alternative products, depending upon whether the proton from C1' or O2' is abstracted first. The former is expected to give rise to 6-pyruvoyltetrahydropterin (PTP) as product, the latter to 6-hydroxymethyl-7,8-dihydropterin (6HMDP), based on analogy with described PTPS (Ploom *et al.*, 1999) and DHNA (Illarionova *et al.*, 2002) reaction mechanisms, providing a possible explanation for the observation of these two products in similar proportions from the PfPTPS reaction. For a Glu residue to act efficiently as a general base catalyst in this way, the pK of the side chain in this environment would need to be raised significantly above its normal level of around 4.3, as seen in other enzymes where Glu has this role (Madhavapeddi and Marsh, 2001).

References for supplementary material

Aspinall, T.V., Joynson, D.H.M., Guy, E., Hyde, J.E., and Sims, P.F.G. (2002) The molecular basis of sulfonamide resistance in *Toxoplasma gondii* and implications for the clinical management of toxoplasmosis. *J Inf Dis* 185: 1637-1643.

Burgisser, D.M., Thony, B., Redweik, U., Hess, D., Heizmann, C.W., Huber, R., and Nar, H. (1995) 6-Pyruvoyl tetrahydropterin synthase, an enzyme with a novel type of active site involving both zinc binding and an intersubunit catalytic triad motif; site-directed mutagenesis of the

proposed active center, characterization of the metal binding site and modeling of substrate binding. *J Mol Biol* 253: 358-369.

Illarionova, V., Eisenreich, W., Fischer, M., Haussmann, C., Romisch, W., Richter, G., and Bacher, A. (2002) Biosynthesis of tetrahydrofolate - Stereochemistry of dihydroneopterin aldolase. *J Biol Chem* 277: 28841-28847.

Madhavapeddi, P., and Marsh, E.N.G. (2001) The role of the active site glutamate in the rearrangement of glutamate to 3-methylaspartate catalyzed by adenosylcobalamin-dependent glutamate mutase. *Chem Biol* 8: 1143-1149.

Ploom, T., Thony, B., Yim, J., Lee, S., Nar, H., Leimbacher, W., *et al.* (1999) Crystallographic and kinetic investigations on the mechanism of 6-pyruvoyl tetrahydropterin synthase. *J Mol Biol* 286: 851-860.

Triglia, T., Menting, J.G.T., Wilson, C., and Cowman, A.F. (1997) Mutations in dihydropteroate synthase are responsible for sulfone and sulfonamide resistance in *Plasmodium falciparum*. *Proc Natl Acad Sci USA* 94: 13944-13949.
